# Operationalising masculinities in theories and practices of gender-transformative health interventions: a scoping review

**DOI:** 10.1186/s12939-023-01955-x

**Published:** 2023-07-27

**Authors:** Julia Zielke, Stephanie Batram-Zantvoort, Oliver Razum, Céline Miani

**Affiliations:** 1 Department of Epidemiology and International Public Health, School of Public Health, Bielefeld University, Universitätsstr. 25, 33615 Bielefeld, Germany; 2 Sexual and Reproductive Health and Rights Research Unit, Institut National d’Études Démographiques (INED), 9 Cr Des Humanités, 93300 Aubervilliers, France

**Keywords:** Gender-transformative, Masculinities, Theory operationalisation, Translating theory to practice, Performativity of theory, Transformation, Health interventions, Theoretical pluralism

## Abstract

Gender-transformative health interventions that involve men and boys are gaining global reach, adaptability to specific geographical, population and epidemiological contexts, public endorsement, and conceptual sophistication. However, the ways in which masculinities are conceptualised and operationalised in theory and practice across these interventions remains unclear. The purpose of this scoping review is to map intervention studies that conceptually grapple with masculinities and analyse: a) how the concept of masculinities is adapted and operationalised in gender-transformative interventions, with respect to intervention population and context, b) what the relationship between the concept of masculinities and its wider theoretical embedding is, and c) on which levels transformation can be observed when working with ‘masculinities’.

We conducted a search in APA Psych Articles, APA PsycINFO, and CINAHL via EBSCO, MedLine, PubMed, and Web of Sciences (December 2021) looking for peer-reviewed studies on gender-transformative health interventions which engaged with masculinities conceptually. There were no restrictions regarding language, publication date, or geography. Forty-two articles were included in this review. Our abductive analysis finds that ‘hegemonic masculinities’ is a central concept in almost all included studies. This shows how the concept is adaptable to a range of different intervention contexts. The review further identifies five theoretical approaches, that help operationalise masculinities on an analytical level: feminist framework, affect theory, critical pedagogy, theories of social change, and ecological approaches. Lastly, this review draws out six levels on which transformation can be observed in the intervention outcomes: relational level, symbolic level, material level, affective level, cognitive-behavioural level, and community-structural level. The discussion underlines that processes and practices of (gender) transformation also require engagement with theories of transformation more widely and advocates for theoretical pluralism. Lastly, implications for practice, including preventative, ecological and community-based care models, are drawn out.

## Introduction

“No health equity without gender equity!” is the underlying assumption of gender- transformative public health interventions, which increasingly gain scope, scale, and sophistication (see for instance [11], p. 9). Gender equity can be understood as the removal of social and economic obstacles to health, thereby giving everyone a fair and just opportunity to be as healthy as possible [13]. Gender transformative interventions (GTIs) focus on unequal gendered power relations as one of these obstacles to health and advocate for equitably distributed benefits among all genders [44]. GTIs go beyond gender-sensitive or gender-responsive approaches that acknowledge and address gendered needs and dynamics but stall at *transforming* gender norms and attitudes in substantive ways. Transforming gender relations on individual, community and structural level can lead towards improved health outcomes in areas like gender-based violence, HIV/AIDS, or sexual and reproductive health and rights [10, 26, 27, 47, 61, 67, 75, 78].

Specifically, GTIs involving men and boys show increasing global reach, adaptability to specific geographical, population and epidemiological contexts, public endorsement, and conceptual complexity [6, 10, 14, 34, 50, 51, 60, 83]. In line with the language used in the intervention literature and for lack of a short-hand, we refer to ‘men’ and ‘boy’ as a relational and fluid gender identity that may be different from their sex assigned at birth and may include queer or non-binary folk. In these types of GTIs, boys and men may recognise traditional masculine gender norms that position men over women, and learn about and practice more gender equal alternatives. Traditional masculine gender norms can be referred to as ‘hegemonic masculinity’ (HM) [21]. The concept refers to a set of beliefs, expectations, actions, and embodied practices such as self-reliance, dominance, self-perceived invulnerability, or emotional control. These are all behavioural patterns that are typically associated with risk-taking and a decreased likelihood to seek help or ‘talk’. Some men report powerful pressures to conform, which in turn may lead to experiences of discrimination, marginalisation, severe stress and clusters of poor health [46]. Dworkin and colleagues [25] add that the onus of change in GTIs with men should not solely lie on men’s willingness to challenge their masculinities, but must also consider geopolitical, economic, and other structural forces.

Yet, there remains a lack of clarity with regard to what kinds of men and masculinities exactly are impacted by GTIs [34]. Feminist philosophers of science and technology have long pointed out the performative potential of knowledge production, insisting that if we change the way we look at things, the things we look at change [9]. The choice of which theories we use to describe the world is thus an ethical one: do our theories reiterate the status quo or do they seek to expose and challenge power inequalities, pointing perhaps towards more just futures? As Jewkes and colleagues [50] observed, theoretically grounded GTIs tend to be more effective, underlining the importance of mapping the heterogeneity of theory at stake in GTIs that engage with masculinities.

With this in mind, this review scopes the literature on GTIs that engage theoretically or conceptually with masculinities, asking how masculinities were employed, developed, and operationalised in theory and practice. A discussion draws out implications for translating theory into practice.

## Methods

We conducted a scoping review that is recommended when a topic has not yet been extensively researched and is of a complex or heterogenous nature [65, 71]. We followed [5] recommended iterative process as updated by Levac and colleagues [59] and the Preferred Reporting Items for Systematic Reviews and Meta-Analysis extension for Scoping Reviews [82].

Prior to and during the research phase, the Cochrane database of systematic reviews and Prospero were searched for relevant reviews or review protocols, ensuring our review focus was meaningfully differentiated from similar reviews in the field.

### Stage 1: specifying research question

Our three research questions are:

1. How is the concept of ‘masculinities’ adapted and operationalised in GTIs, with respect to intervention population and context?
2. What is the relationship between the concept of masculinities and its wider theoretical embedding?
3. When working with ‘masculinities’, on which levels can transformation be observed?

### Stage 2: identifying relevant studies

**Fig. 1:**
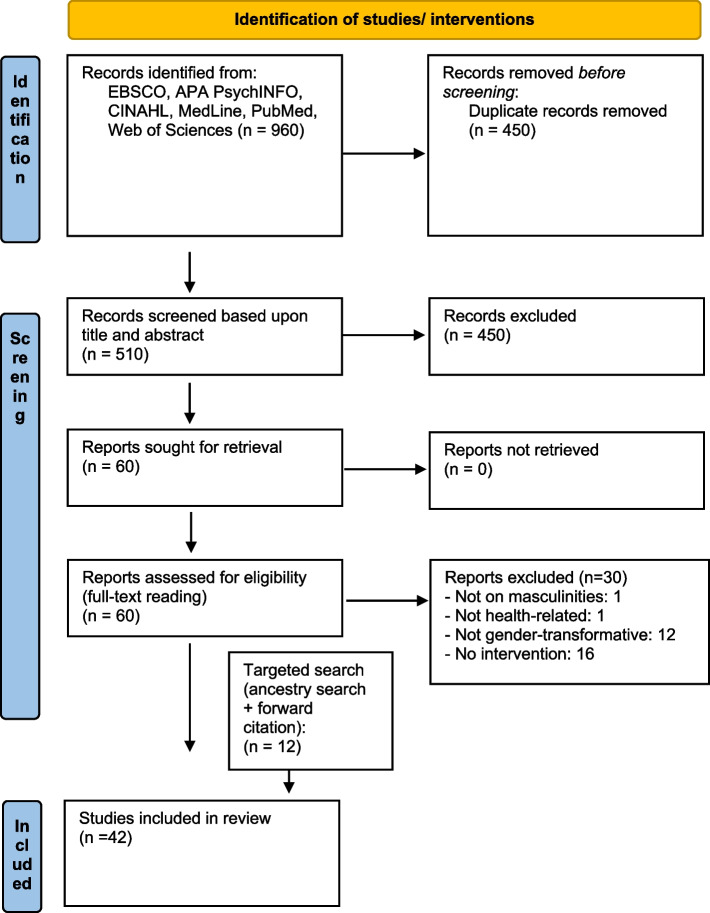
Prisma flowchart. Adapted from: Page MJ, McKenzie JE, Bossuyt PM, Boutron I, Hoffmann TC, Mulrow CD, et al. The PRISMA 2020 statement: an updated guideline for reporting systematic reviews. BMJ 2021;372:n71. https://doi.org/10.1136/bmj.n71

We searched the following electronic databases between December 2021 and January 2022: APA Psych Articles, APA PsycINFO, and CINAHL via EBSCO, MedLine, PubMed, and Web of Sciences. A sample search strategy of PubMed is provided in Appendix 1. Ancestry searching and forward citation tracing helped identify additional source. Details on identification, screening, inclusion and exclusion of literature can be identified from the PRISMA flowchart in Fig. 1.

### Stage 3: study selection

We included any descriptive, qualitative, quantitative, or mixed-method study as well as frameworks on interventions where a) the intervention is gender-transformative, b) there was an explicit engagement with men and masculinities on a theoretical and/or conceptual level, c) the intervention was health-related, and d) the study underwent peer-review. Interventions were classed as gender-transformative if the authors of the respective study explicitly describe at least one part of the intervention (framework, design, implementation, or outcome) as gender-transformative [16]. Interventions were included when engaging with theories and concepts on men and masculinities by offering a structured explanation on the relation between ‘being a man’ and issues surrounding gender inequity [7]. We included papers published in any language as long as the abstract was available in English, published at any time, in any region or place. Retrieved papers published in a language other than English or German were translated through the online software DeepL (www.deepl.com). Studies were not critically appraised due to their heterogenous nature, which is standard practice in scoping reviews [70].

Following PRISMA guidelines (see Fig. 1), in the first screening stage (title and abstract screening) two researchers (JZ and SBZ) independently screened half the titles and abstracts based upon pre-defined in-and exclusion criteria (see above), after having compared screening outcomes of a cross-sample of 20% of the retrieved articles to ensure consistency. In the second screening stage (full-text reading), both JZ and SBZ read all remaining articles in full-text, and disagreements were discussed with CM. The relatively high number of excluded reports (*n* = 30) can be explained as research questions were refined after first screening stage.

### Stage 4: charting the data

In line with the research question, we iteratively developed a data extraction sheet in Excel. JZ and SBZ ran a sample extraction of six studies. After some alterations to the extraction categories, SBZ, JZ and a student research assistant extracted remaining data. Two reviewers (JZ and CM) checked for accuracy. Extracted data included: bibliometric study identifiers, study population and location, health-related area, objectives of study, methods of study, theoretical or conceptual considerations of study, outcomes/ findings of study, design, aims, and components of intervention, theoretical approach or conceptualization of men/ masculinities, operationalization of theory in intervention.

### Stage 5: collating, summarising and reporting the results

All included studies were grouped into their respective interventions and a narrative synthesis conducted across each intervention [5]. For data on conceptualisation of masculinities, JZ used abductive analysis that seeks to hold a middle ground between deductive and inductive forms of analysis and pays attention to similarities and differences between different theories and the paradoxes, conflicts and puzzles that may arise between different explanations [28, 48].

## Results and findings

A total of 960 studies were screened and 450 duplicates removed. The relatively low number of 960 can be explained by the fact that our specific search terms a) do not have too many sensible synonyms and b) are usually not used in discourses outside our specific review context. The remaining 510 studies were screened for title and abstract. Of those, 60 matched the inclusion criteria and were moved to the full text stage; here 30 were excluded and 30 included. In addition, a targeted search was carried out and key publications such as Ruane-McAteer and colleagues review of reviews on masculinities in sexual and reproductive health [75] or MacArthur’s overview of gender-transformative approaches [60] were studied carefully for additional references In the targeted search, ancestry searching and forward citation tracing helped identify another 12 documents. Additional queries amongst key scholars in the field and informal queries in Google Scholar and Google yielded no additional data.

Data was extracted from 42 articles that span 16 interventions. One larger intervention led by the NGO Promundo was adapted in various different contexts across the world, some of which are known under a different name. If these were to be considered separate interventions, we count 21 different interventions in total.

### Characteristics of intervention

Table 1 shows all included studies, sorted by intervention.

**Table 1:** Characteristics of included studies

Name of intervention and acronyms used in this review	Literature	Short description of intervention, including area of health and population	Location	Theoretical engagement with masculinities	Components of interventions that operationalise masculinities (summary)	Outcomes or findings in relation to intervention aim
Boys to Men (BTM)	[55–57]	Intervention with groups of disadvantaged young men and boys to challenge harmful gender norms with view to gendered violence and relational well-being	USA	Relational accountability, reflexive practice for gender/social justice, intersectionality, feminist framework and pedagogy of empathy based on Freire	18-h small group discussions by trained community members to support young men and boys in ethical self-reflection helps in emphatic ways; content focusses on raising awareness of restrictive masculinities and how this shapes identity, emotions, sexual consent, and violence	Qualitative findings highlight role of facilitators in developing empathy and affective intensities; facilitators also grow as part of the shared journey; general focus on the importance of affect in transformative work
Coaching Boys into Men (CMIB)	[62, 64]	Violence prevention programme that teaches sports coaches to talk to their male athletes about non-violence, respect and healthy masculinities thus fostering bystander intervention	USA and India	Social norms theory and other theories of social influence; no HM	Training course for coaches to teach them about how to help athletes adapt positive masculinities, call out gender-inequitable behaviour, and foster bystander intervention	3 months post intervention evaluation indicates more bystander intervention, less dating abuse, more gender-equitable attitudes and a general appreciation of meaningful conversations with coaches
First batterer intervention program	[76]	Intervention aimed at men convicted of gender-based violence with the aim to conform participants to ‘good manhood’ and prevent re-offending; intervention participation was a choice participants made over additional prison sentence	USA	HM, additional focus on social norms theory, rational choice theory, and interactionalist approach	Facilitator-led group discussions over six months that confront men with right choice to make which helps them take appropriate actions in accordance with ‘good manhood’, including reflections on feeling and acceptance of aggressorship	Ethnographic study demonstrates that programme failed in its intention to have men take full responsibility of their action, and adapt a pro-feminist masculinity; men learned egalitarian rhetoric but might have been mere lip service
Machofabriken	[50, 51]	Interventions to reduce violence and foster positive health behaviours, aimed at mixed-gender young people in schools and leisure activity clubs	Sweden	HM with addition of hypermasculinity, gender systems theory, poststructuralism, queer theory, intersectional theory; study focusses on how HM is operationalised	Reflection on male privileges and patriarchal order through short films and subsequent association exercises that help translate critical challenges to HM into everyday practices	n/a
Men’s Story Project (MSP)	[68, 69]	Social and behaviour change communication initiative based on public sharing of personal masculinity narratives to prevent gender-based violence	USA and Chile (as well as other planned locations)	HM with additional focus on social constructivist and intersectional characteristic; social cognitive theory	Public live performances of diverse personal masculinity narratives through prose, poetry or music demonstrating cost of HM; operationalised through Transportation-Imagery Model which helps men “transport” themselves into someone’s point of view and shift entrenched views on, e.g., sexism or homophobia; intervention easily scalable movement-building	Focus groups highlight building safe spaces, challenging stereotypes and prejudices, empowerment and healing, reinforced commitment to end violence, raised awareness of gender justice and various, intersectionally aligned masculinities; building of empathy, positive role modelling and spreading of reflections outside intervention context
Reducing Sexism and Violence Program	[8]	Violence prevention programme for Middle School boys	Maine, USA	HM with additional focus on Pleck’s gender strain model, positing that (non-)adherence to traditional masculine norms causes acute stress and anger in men and violence is a way to regain social dominance	Four one-hour sessions that foster critical thinking about rigid gender norms and bystander intervention	Randomised control trial showed significant decrease in treatment group in tolerance of violence; but neither control group nor treatment group showed a change in norms supporting violence
Soils, Food and Healthy Communities (SFHC)	[58]	Participatory nutrition education intervention that tackles child malnutrition, focussing on intra-household relationships	Malawi	HM and critical men’s studies; postcolonial feminist framework with participatory approach	“Recipe days” as participatory activity to promote healthy feeding, share skills and encourage gender-equitable household relations using dialogue approach	Qualitative research shows more male involvement in care and household work, including cooking; improved knowledge of nutrition and its link to gender roles amongst whole community; defining strategies for new, emergent masculinities
The Young, Black Men, Masculinities, and Mental Health (YBMen)	[86, 87]	Social-media-based intervention aimed to improve the mental health of young, black men	USA and online	HM with focus on what this means for intersectional challenges of young, black men; role of (online) social networks is highlighted in understanding how masculinities are shaped and changed; intervention design engages with social cognitive theory, theories of social network and support and social network approach	Gender-sensitive, age-appropriate and culturally-sensitive educational messages around black men’s mental health on Facebook and Instagram with group activities scheduled over six weeks	n/a
Unnamed “diploma course”	[4]	Peer- education course for civil servant aimed to prevent violence against women	Medellin, Columbia	HM with additional focus on intersectionality and Freire’s transformative pedagogy	100 h training course based on peer support (men educating men); built on encounter, belonging and subjective responsibility for shared knowledge creation within everyday spaces; course content covered gender norms, sexuality, violence, health, (verbal) violence, bullying at school and general awareness raising	Mixed method evaluation finds that a change in understanding of masculinity happened on practical, cognitive and affective level; students highlighted improved relationships as central achievement
Unnamed intervention for perpetrators of violence	[12]	Prison-based intervention with a feminist collective that works with male perpetrators of violence against women	São Paulo, Brazil	HM with feminist framework and focus on minority standpoint theory	16 group discussion meetings with feminist collective that aimed to increase critical reflection through dialogues	Ethnographic study found that men might have learned to play along with “feminist language games” but continue to perceive themselves as victims rather than perpetrators; norms remain unchallenged although behaviour might have changed
Unnamed intervention operated by “Valley Trust”	[52]	Livelihood intervention with men in poor, rural neighbourhood with a view to prevent HIV/AIDS and improve sexual and reproductive health	South Africa	HM	Group meetings which focussed on land-use and other concrete challenges men faced; participatory workshops about household roles	Qualitative case study describes the creation of safe space to share experiences and worries about poverty and learn about HIV; focus on land-use allowed men to be active in provider role, affirming a positive aspect of traditional masculinity
Unnamed intervention in Italian Prison	[80]	Prison-based intervention with male perpetrators of violence against women	Italy	HM with focus on links to criminality and cultural elements of Italian culture, like Madonna and whore dichotomy and specific role and relationship to (their) mother	Arts-based methodology that includes psychodramatic techniques and photo-based therapy	Interviews showed that intervention only partially achieved objectives as men shifted towards a more benevolent sexism; possible factors include that prison culture reinforces macho behaviour based on a code of honour
WiseGuyz	[29–32]	Participatory healthy relationship promotion programme for Grade 9 boys, aimed to improve mental and sexual health and reduce male-perpetrated violence	Canada (primarily urban)	HM with focus on marginalised masculinities; intersectional feminism; and various theories of social influence including: capability, opportunity, motivation and behaviour model; information-motivation behaviour model; theory of social influence; role of (ethnocultural) friendships highlighted	20 sessions develop through school-community partnerships over one academic year, content on: healthy relationships; sexual health; gender, sexuality, and media; and advocacy and leadership	Various quantitative and qualitative photo-based evaluations point towards improved mental wellbeing, improved friendship quality, enhanced emotional expression through creation of safe spaces, ability to identify social support, improved capabilities to resist adhering to stereotypes, and a general shift in masculinity norms
Stepping Stones/ Creating Futures	[37, 38, 40]	Participatory behavioural and structural intervention aimed to reduce gender-based violence and HIV, reduce substance abuse, and improve overall sexual health	South Africa (various urban, informal settlements)	HM with focus on hypermasculinity; and Freire’s transformative pedagogy	21 three-hour sessions (e.g., group discussions, role-play, or body mapping) on skills building, critical thinking around gender equality, communication, safer sex, and livelihoods based on building personal responsibility and critical consciousness on collective level	Studies using longitudinal in-depth interviews with men and their partners, focus groups indicate importance of safe spaces for critical reflection and vulnerability thus building alternative masculinities; men engaged more with formal and informal economy on basis of intervention; unclear if masculinities were radically shifted, outcomes perhaps more modest shift to less violent but still hegemonic masculinities
	[39]					Randomised control trials across various urban settlements indicate significant reduction in violence and alcohol consumption; economic benefits with significantly better earnings for women
	[41]					Ethnography with focus on facilitator role shows centrality of emotion in their work, facilitators position themselves as ‘successful men’ that participants tried to emulate e.g. by establishing condom use and consent; unclear if change was merely behavioural with no shift in norms and attitudes
	[36]					Post-hoc analysis of RCT followed latent class analysis and reports that intervention has greatest impact on men in most violent class (hypermasculine men associated with gang membership, economic hardship, and adverse childhoods)
One Man Can (OMC**)**	[18]^a^	Intervention with various foci on improving men’s relationship, reducing spread and impact of HIV/AIDS, and reduce violence against women, children and other men	South Africa	HM and wider consideration of gender norms on community level with focus on roots and causes of violent behaviour, including childhood trauma	Workshops on six topics: gender and power, critical reflections on the norms and practices associated with hegemonic masculinity, gender and violence, gender and HIV/AIDS, healthy relationships, and taking action for social change. The workshops are facilitated by men and are held in groups of fifteen to twenty. The sessions provide ample space for men to reflect upon human rights, women’s rights, and how masculinities are defined, practiced, reified, and can be challenged in relationships, communities, and broader society	‘Local cultural’ view upon gender norms and ‘human rights’ were not only perceived as naturally given, but also contrasting each other as being in an antagonistic relationship. OMC efforts to establish a human-rights perspective upon gender by gently giving space for discussion, providing information and critical self-reflection; yet recognizing that gender-transformation remains a long-lasting and ongoing process
	[26]					60 in-depth interviews focussing on masculinities, gender relations and rights, violence, gender and HIV risk, alcohol, fatherhood, and relationships reveal how men reconfigured notions of HM in terms of beliefs and practices in relationships, households, and women’s rights
	[33]					60 in-depth interviews fosucssing on HIV, HIV- related stigma, attitudes about HIV testing, care, and treatment, and how OMC changed beliefs and behaviours related to the HIV treatment cascade showed an increased capability to overcome masculinity-related barriers to testing/care/treatment. Interviews revealed an increased ability to express vulnerability and discuss HIV openly with others and greater willingness to be tested for HIV/ receive HIV care and treatment
	[45]					53 in-depth interviews with men shows that men reduced their alcohol intake and improved partner communication (e.g., sexual decision making)
	[81]					Longitudinal interviews report positive change in gender-equitable norms and reduction in violent behaviour, most notably amongst community mobilisers. Some participants struggled to implement non-violence in life, suggesting that more needs to be done for men with history of violence
	[84]					60 in-depth interviews revealed changes in fatherhood beliefs and practices due to the participation in OMC (shifts in parenting style from financial ‘providership’ role to one of increased involvement, companionship, nurturing, and affection). Several fathers described a shift towards being less violent and being more caring and protective
	[79]		Uganda	HM, including a focus on Greene’s (2006) model of engagement (men as clients, men as partners, men as agents for change)	Health education through various community channels and engagement with stakeholders, like churches; training of 120 peer educators who created safe spaces for peer learning	Surveys and focus groups show men reporting greater understanding of sexual and reproductive health and more equitable decision making in household; degree of change remains unclear
Promundo: Program H/ Manhood 2.0/ Men Care + / Young Men’s Initiative/ Yaaro Dosti/ EngenderHealth	[1, 53, 64]	Manhood 2.0 extends Program H by addressing social media use (e.g., internet pornography) and taking an intersectional stance through examining privileges (e.g., white privilege) and practicing bystander skills, aiming to reduce sexual and intimate relationship violence and enhance gender equitable practices with socially disadvantaged African American adolescents	Pennsylvania, United States	HM and ‘rigid masculinity’, including Social Norms Theory Theory of Reasoned Action and Heise’s Framework of Gender and Power	Design of group-Intervention sessions that include discussions on critical analysis of internet pornography, exploration of intersectionality by sharing experiences of racism and marginalization, including an examination of white privilege and male privilege through art based-activities, information on sexual health, scenarios on bystander interventions	n/a
	[66]	Young Men’s Initiative changes dominant masculine ideals that underlie violence, sexual risk-taking and other unhealthy behaviours for adolescents attending vocational schools	Bosnia and Herzegovina, Croatia, Kosovo and Serbia	HM	The programme included 21 modules organised into four content areas (gender, violence, sexual and reproductive health, and substance use). Workshops utilised participatory and interactive techniques (e.g. role play, group brainstorming, games, etc.) and emphasised asking provocative questions and creating space for boys to reflect on issues themselves	In-depth Interview and focus-group discussions reflected an appreciation of multiple masculinities and a growing ability to recognise gender norms as socially constructed and changeable
	[17, 85]	Yaaro Dosti (friendship) promotes gender equity, prevents gender-b	India	HM	The intervention started with an intensive week of group education activities, facilitated by both peer leaders and gender specialists, followed by 2–3 h sessions every week, led by peer leaders, over the six months. Main themes: STI/HIV risk and prevention, partner, family and community violence; gender and sexuality; and reproductive health. The group educational activities were based on participatory methods of learning with extensive use of role plays, games and exercises that engaged young men in discussion, debate and critical thinking	Pre-and post-intervention survey and qualitative interview indicate positive changes in attitudes towards gender, sexuality and intimate relationships
	[24, 54]	MenCare + addresses sexual, reproductive and maternal health, aims to reduce harmful gender norms, and promotes fatherhood involvement for young men and couples	Rwanda and South Africa	HM	Group education on sexual and reproductive health and rights and equality for young men and women, and on maternal, newborn and child health and caregiving for fathers and mothers. Groups were accompanied by community-level campaigns to change social and gender norms. Partner organisations worked with health services to improve the quality and accessibility of sexual and reproductive health services	Randomised control trial (couples) showed substantial improvements in women’s experience of physical and sexual intimate-partner violence, contraceptive use, and partner support during pregnancy. Men’s dominance in household decision-making and improvements was significantly reduced. Young men’s gender equitable attitudes increased significantly post-intervention. The use of health care services by young men regarding sexual and reproductive health issues did not increase. However, more men did report feeling comfortable asking a health care professional for information about sexuality related issues
	[72, 73]	EngenderHealth tackles the fields of HIV and gender-based violence preventions for young male factory workers and vocational students aged 15–24 years	China	HM	Eight participatory educational sessions focused on gender, sexuality, relationships, HIV/STI (sexually transmitted infection) risk, and violence	Quantitative and qualitative findings indicate that positive changes in gender-related attitudes, gender-based violence, and partner communication for both school and workplace setting in China

^a^This study is a comparison of three interventions, of which only one matched our inclusion criteria

The health-related area most frequently addressed by the interventions was gender equality in a wider sense. More specifically, this included gender-based violence prevention (e.g. sexual or dating violence, intimate partner or domestic violence, femicide, or a reduction of harmful gender norms such as male dominance, sexism, or homophobia) [1, 4, 8, 12, 17, 18, 23, 24, 26, 29–32, 36–40, 50, 51, 53, 55–58, 62, 64, 66, 72, 73, 76, 80, 81, 84, 85], child (mal)nutrition [58], healthy masculinities [68, 69], mental health [86], sexual and reproductive health (e.g., STI and HIV/AIDS prevention) [18, 24, 26, 33, 36–40, 52, 54, 72, 73, 79, 84], a syndemic cluster of alcohol use, violence and HIV/AIDS [45], or fatherhood involvement and positive parenting [24, 81],van den [84]. Geographically, the interventions were located in Africa (*n* = 9), North America (*n* = 8), Asia (*n* = 5), South America (*n* = 3), Europe (*n* = 3) and Australia (*n* = 1). Regarding the target population, interventions included a variety of differently situated men, whereas only few interventions additionally addressed women (usually the female partners of participants). Boys and adolescent men up to 18 years old were addressed in nine interventions, whereas the age group 18–35 was involved in most interventions (*n* = 12). Only three interventions included men aged older than 45 years and seven interventions did not report the age of their participants, most of them being community-based interventions. Moreover, seven interventions were run as community-based projects, all of them in deprived, poor, mostly rural areas with a high prevalence of violence and HIV. Participants were recruited from schools, youth-clubs and sports-clubs in six interventions. Four interventions approached their participants through their workplaces or reported on men working in specific areas. Participants of two interventions were currently imprisoned or accused perpetrators of violence against women. Interventions involved different components, ranging from participatory workshop or events to group education sessions, and community actions.

Additional findings are structured along our three research questions.

### How is the concept of ‘masculinities’ employed and adapted in GTIs, with respect to intervention population and context?

On a general level, we can observe that the concept of hegemonic masculinities (HM) is featured in all gender transformative intervention (GTI) studies to various degrees and depths except for one [62, 63]. Engagement with HM ranges from a mere citation of one of Connell’s [20] or Connell and Messerschmidt’s relevant publications [21], to a page-long critical discussion. Studies usually include a generic definition of HM, describing it as a culturally ‘ideal’ or superior version of manhood founded on heterosexuality and dominance.

Apart from or in addition to HM, authors introduced various other ‘types’ of masculinities. These included terms like ‘inscrutable’ masculinity (Morrell cited in [52]; characterised by a containment of emotion and unwillingness to talk about them. As a member of the focus group puts it: “often you only find out that a man is sick if he is lying on the floor and not moving” [52], p. 237). Another ‘type’ is hypermasculinity or youthful masculinity which emerges in poverty-struck and trauma-struck communities, where violently defending one’s honour against other men and having numerous sexual partners is seen as traditionally manly, while economic provision is much less important (see for example: [37, 39, 62]. It is within this poverty-struck context that Kerr and colleagues [58] report a ‘crisis of masculinity’, where men struggle to uphold hegemonic ideals leading to fractured, hybrid, contradictory and shifting masculinities, that centre on male control, power and violence. At the same time, this is also opening up opportunities for emergent forms of masculinities.

Most studies also stress the relational and fluid character of masculinities and the fact that masculinities are not a universal category but emerge and are shaped by their specific local intervention contexts. YBMen [86, 87], for instance, highlights the role of ethnicity and remarks that black culture tends to have unique expressions of both masculinities and mental health. As a consequence, mental health challenges for young, black men remain unseen and misunderstood. Similarly, an intervention in South Africa highlights the specific challenges that Zulu men face. Here, local derogatory terms like “Uskhotheni”, translated as “a man who does not work, someone who should stay in the bushes because they are useless” [52], p. 237) indicate the detrimental effects South Africa’s economy has on the destabilisation of traditional masculinities. Societal expectations to marry young and having multiple sexual partners, as described in an intervention in India [85], shows how taking care of one’s family and sexual potency was a key factor to feeling like a ‘real man’. Another intervention in Brazil [12] show how legal and legislative factors, in this case the recent enforcement of the Maria de Pehna Law, a law persecuting offenders of domestic violence, puts strong societal pressure on violent expressions of *machismo*. The importance of culture is exemplified in an intervention in Italian prisons with perpetrators of femicide [80] which remarks that Italian culture uniquely shapes men’s attitudes towards women. As an example, the authors mention the Madonna and whore dichotomy and, taking a psychoanalytic lens, the strong presence of the mother within Italian families—all of which need to be carefully taken into account when dismantling the prisoner’s culturally influenced gender norms. Culturally mediated threats to losing one’s face or being ridiculed in one’s community were exemplified in an intervention on child malnutrition, where men felt it was unmanly, awkward and shameful to be involved in childcare [58].

The above examples show the importance of a) *identifying* the various factors that shape and are shaped by the respective masculinities at stake in the specific intervention contexts and b) designing and implementing an intervention that *responds* to these various structural factors. A good example of how this is done is Promundo’s Program H [17, 66, 72, 73, 85], a GTI with wide global reach and includes adaptations like Manhood 2.0 [1, 53, 64] or MenCare + [24, 54]. A key component of these interventions is a formative research stage that entails a mapping of existing community infrastructures and assets, followed by workshops with stakeholders and a community consultation. The scripts for facilitators and programme content are adapted according to the findings of the research. As an example, formative research for Manhood 2.0 —set in a disadvantaged, primarily African American neighbourhood in Pittsburgh, USA— highlighted the ubiquity of social media use amongst young men, the critical role of Internet pornography in shaping gender norms, and the need for an intersectional understanding of privilege.

### What is the relationship between the concept of masculinities and its wider theoretical embedding?

Although, as a concept, HM might be adapted to reflect local histories and cultural traditions [50, 51], it may not be sufficient in explaining multi-facetted mechanisms for change on a theoretical or analytical level in full. This was evident in our data in which we identified five key theories that support, galvanise, embed, and otherwise help operationalise masculinities on a theoretical and analytical level.

#### Feminist frameworks

Several studies mention or build on important groundwork by queer, intersectional, poststructuralist, and feminist scholars [15, 22, 77]. These have laid the foundation for a wide body of scholarship on masculinity by highlighting how social norms are socially constructed and how identity is fluid and relational. Feminist scholarshop also underlines the ethical importance of care and reflexivity and argues that visibility of minority standpoints is important in advancing gender justice and social justice (see for instance [12, 49–51, 57]. Other authors [58] take an explicitly feminist postcolonial stance to reveal, understand and challenge unequal power relations in a rural agrarian context in Malawi, a place with a history of slave and ivory trade. Grounding work with men in feminism is especially crucial as organisations like Sonke Gender Justice (van den [84] are cautious not to be perceived as an organisation that empowers men or champions men’s rights. Instead, they seek to empower women and improve their health by engaging men in change-making,thus reaping benefits for all.

#### Affect theory

With a firm footing in feminist thought but a theory on its own, affect theory stresses the role of emotions in responding to our (social) environments. Affect theory is key when thinking about how being confronted with challenging intervention content. This might include homophobia, violence and entitlement. In this context, participants and even facilitators report to feel angry, emphatic or self-conscious (for example in [4, 31, 32, 55, 56]. As one participant in a study in the Young Men’s Initiative (also based on Program H) noted after participating in an exercise that lined up two groups facing each other, with one group being the ruler and the other following their commands as servants: “and then facilitator asks, ‘How do you feel?’ … It kind of left an impression. You really see how it is when someone rules over you and what is the feeling when you rule over someone.” (in [66], p. S212). WiseGuyz also stresses the importance of emotional freedom that may develop through close friendships that can help people break out of hegemonic norms and freely and safely explore what masculinity might mean to them. Building capacity for emotional expression and vulnerability seems to be an important factor in transforming masculinities in individuals. Affect is also key in critical pedagogy approaches; emphasising here that you can only know different if you can feel different [55].

#### Critical pedagogy

Critical pedagogy is an approach to transformative education championed by Paolo Freire [35] which highlights the role of critical consciousness building as a collective practice in recognising, speaking about and critiquing various injustices. Its aim is to take action, empower each other and end oppression. With this ambition, critical pedagogy naturally lends itself to gender- transformative endeavours and was a cornerstone in several participatory interventions that wanted its participants to reflect more critically on masculine norms, e.g. in Program H, BTM or Stepping Stones (see for instance [36, 37, 39, 41, 53, 55, 58]. Operationalising masculinities in a way that transforms norms also means that participants have to learn about the ways in which gender and class structures are historically constructed, specifically they also have to learn what the cost of hegemonic masculinities are for individuals and society at large. This learning is not a purely cognitive exercise of absorbing knowledge, but rather requires the participants to build critical consciousness; to be “touched in their being, not only knowing and doing” [4], pp. DeepL Translation, n.p.). This way students may become teachers themselves, grow personally, become agents for change and help end stigmatisation. Freirian pedagogy does not come uncritiqued, however, and as Gibbs and colleagues [37] remark, interventions may just help men adapt to changes in their relationships rather than pushing for radical social change on a holistic scale. Facilitators also often deviated from participatory scripts to instil important messages about consent in didactically more conservative top-down manners.

#### Theories of social influence

The term ‘theories of social influence’ groups a heterogenous body of theory like social norms theory [31, 32, 53, 62], interactionist approaches leaning on Goffman [76], social cognitive theory according to Bandura [68], or theory of reasoned action [53, 62]. Broadly speaking, these theories highlight the role and function of community in shaping and challenging social norms. Role models and bystanders may play an importance part in these processes. These theories stress that (non-)conformity to norms is not just happening within the individual (who needs to assess and reflect on the consequences and costs of their gender attitudes and behaviours) but is equally influenced by how others perceive and judge their attitudes and behaviour (and how their judgment makes the individual feel and react).

The transformative aspect here is that, according to theories of reasoned action [53, 76], individuals have a certain – albeit at times structurally limited – degree of rational choice as to which masculine privileges, values, beliefs and norms they want to internalise, assert and enact in social interactions with others. This analytical perspective is useful when operationalising masculinities in more narrative-based interventions that seek to foster self-efficacy, i.e., the capability to reliably embrace gender-equitable masculinities and to resist social pressure to conform to more hegemonic masculinities beyond the intervention context [68].

#### Ecological approaches

Ecological approaches to masculinities put greater emphasis on the socio-spatial aspects that determine which kinds of wider structural environments might be conducive to societal change and personal transformation [32, 37, 66]. Campbell and Cornish (cited in [37], p. 504), for instance, highlight three interlocking spheres in this regard: the material-political, the relational and the symbolic. These approaches shift the locus of change away from the individual man and onto entrenched societal-level inequalities that hinder or enable men’s agency in specific ways. Examples may include the South African apartheid, income inequality, political culture and their media landscapes, wars, and migratory needs [25].

Likewise, a livelihood intervention focussed on agriculture and household roles in the context of child malnutrition [58] is based on an ecohealth model which explicitly highlights the interrelationships between ecological, social, and economic factors in addressing human health. The authors propose that emergent masculinities ought to be embedded into broader agricultural-nutritional concerns in a culturally respectful and sustainable way.

### When working with ‘masculinities’, on which levels can transformation be observed?

This review identified five levels or spaces which triggered a shift in masculinities and show how transformative processes are operationalised in practice. We have looked at intervention components and reported outcomes to assess this question.

#### Relational level

The first level concerns relationships which we can separate into two sub-categories: a) relationship with intimate partners and children and b) relationship with a wider social network.

##### Relationship with intimate partners, with children and role in the household

Interventions lead to more gender-equitable norms in comparison to before the intervention participation. Interventions can shift concrete views, for example in relation to men’s justification of using violence against partners and children [45], their emotional involvement as fathers (van den [84], joint decision making and condom use [85], share of cooking duties [58], or a general appreciation of what it means to be vulnerable in front of one’s family [55]. As perceptions of masculinity shift, so too does the role men take with their partners and children. As one 33-year-old OMC participant reported:

*OMC changed a lot in the relationship I have with my child because the bond I developed became even stronger. OMC taught us to show the love and affection we have for our children instead of seeing ourselves as providers.* ([84], pp. 119-120)

This was also communicated by their respective partners, who reported in Stepping Stones that men were, overall, less violent towards them and that they had a chance to earn some money on their own [38–40].

Such reports were not uncommon and also applied to the relationship with romantic partners, as another 23-year-old OMC participant shared:

*At the training we were advised on how to treat our girlfriends and about the importance of treating them well[… ]I liked what was being said there so I decided to apply it to my life and I am seeing a difference in the way I am treating my girlfriend.* ([26], p. 191)

In the SFHC intervention, a 33-year-old female study participant reported similar improvements in terms of support and autonomy:

*I’m now able to ask my husband to look after the child at home while I am doing some work. Previously, I couldn’t even try to request that because I was afraid he could complain to our marriage counsellors who could fault me. But now, he knows that his case can’t be taken seriously because the counsellors also learnt the importance of gender during the recipe day [a part of the intervention] *[58]*, p. 9)*

Relationship with wider social network and other men.

Outcomes also show significant shifts in men’s relationship with other men and their wider social networks, specifically regarding challenging stereotypes about non-hegemonic men. This is also exemplified in this quote by a participant in an adaption of Program H in Eastern Europe:

*Before I came to the advanced training, I didn’t have that opinion [that I could be friends with a gay person]. I have made friends with such a boy. I would never have done that before, because on the one hand, I was afraid of him, and on the other hand, I was afraid people would say that I was gay, too. But today, I think Be a Man has changed my opinion completely.* [66], p. S215).

Building understanding, empathy, and solidarity with other men was also evident in OMC [81] and the Men’s Story Project, where upon hearing a man share his experiences of parental abuse, a participant felt less alone in his experience of the same thing:

*“I was empowered, I gained […] community. It corroborated my feelings, my concerns around masculinity. I had never talked to anyone about masculinity, except maybe some female friends. Being with a group of men, talking about it - I’m on the right track…“* [69], p. 9)

The importance of friendship was also a key building stone in WiseGuyz [29–32], positing that an improvement in friendship quality between “ethnocultural boys” allows for intimate disclosure, emotional support and improves overall wellbeing.

The importance of community cannot be underestimated in shifting gender norms: in a randomised control trial, Miller et al. [64] report positive shifts in gender norms in both treatment and control group, hinting at the fact that it might have not been the specific content of the intervention but rather the feeling of belonging to a community that has a positive impact on gender-equitable attitudes.

Interestingly, not only men’s relationships improved in the interventions. Peretz and Lehrer [68] for instance show how women attending an intervention activity reported a profound shift in their understanding of lived realities of black and disabled men. One woman reported how because of the intervention she now felt less threatened by two African-American men who walked behind her one day [68], p. 12).

Although perceptions of marginalised masculinities may positively shift and social networks may widen and change, participants also reported that they struggled to perform non-hegemonic masculinities outside the intervention and in their wider social circles and feared to be ridiculed or excluded [12, 52, 80]. The challenges of stepping away from bad relationships and friendships also become clear in the testimony of a Stepping Stone participant:

*Friends are very hard to get rid of when you are trying to change, because they are always around drinking and you find yourself drinking with them, but I tried to resist and avoid bad friends*. [37], p. 511)

These structural barriers to a more radical transformation of gender norms were identified in other interventions, too, especially those working with offenders of gender-based violence and prison populations [12, 76, 80]. Here, studies show that the intervention might have only given men a language to *appear* more equitable while their beliefs and attitudes about gender roles as well as ‘good’ and ‘bad’ femininity and masculinity remained largely unchanged. These interventions were successful in reducing violence, as the costs appeared too high to the men, but, at the same time, unsuccessful in that they merely shifted men towards more benevolent sexism.

#### Symbolic level: making available alternative representations of masculinities

Transformations on the symbolic level centre around the availability and accessibility of different representations and ideas of masculinities within everyday interactions and media representation. They try to understand which specific representations and symbols people draw on in their understanding of masculinity (Campbell and Cornish in [37]. Some interventions worked with already existing versions of masculinity and tried to focus on and slightly reframe positive aspects of masculinities, such as “men as breadwinners” [52]. In Stepping Stones, CMIB, Men’s Story Project and some versions of Program H, on the other hand, facilitators and practitioners took the part of positive male role models that emulated success without abiding to traditional male gender norms; thereby offering an alternative to existing symbolic representations. These ‘aspirational masculinities’ [66] included an acceptance of being vulnerable and empathic, and being aware of one’s privilege and communicating openly, all traits usually not associated with hegemonic ideal of manhood [86]. Interestingly, some of these narratives around aspirational or successful masculinities framed transformation not in terms of self-reflection but in terms of redemption and salvation ([39], p. 547).

In the Man’s Story Project, participants were able to find a way to change the view they have of themselves, realising that, even though they have been violent in the past, they are not demonised as men. As one participant in the intervention states:

*“For me, it was like, “Finally, someone understands.” Some of the women that came up [after the presentation] - those were the women that I felt like I could never get to understand what it was like to be a violent person, to be angry… And then for them to give me this response, “I understand” - it’s like finally, you know… I’m not just stigmatized or demonized as this horrible person who’ll never heal…”* [68], p. 10).

However, as Gibbs and colleagues remark [36], despite developing wider symbolic representations of what being masculine might entail, some elements of dominant masculinities like heterosexuality and control were not problematised and in some instances even celebrated.

#### Material level: impacting material conditions

Material impacts were evident in interventions with a component on livelihoods and economic opportunity [37, 38, 40, 52, 58] and make sense considering that violent masculinities sometimes develop out of childhood adversity and poverty [64]. In OMC, for instance, participants learned budgeting tricks and CV writing, which helped participants secure jobs in the formal and informal economy. Although as Gibbs and colleagues [37], p. 509) remark these efforts should not be over-interpreted as even a perfect-looking CV does not secure jobs in an economy where there are simply no jobs, meaning that men continued to be tied into exploitative labour conditions.

In SFHC [58] so-called ‘recipe days’ played a pivotal role in helping couples understand the value of healthy eating in child development. In turn this meant that husbands were allocating (more) household resources to their spouses so that they may prepare nutritional food for their families. A 38-year old female participant shared that her husband apologised to her after the recipe day and “that he was not aware that he was contributing to ill health of our family when he was monopolizing the resources” [58], p. 9). In another unnamed intervention [52], men participating in a programme aimed at reducing HIV rates felt that they had to compensate their perceived (economic) uselessness through dominance and violence. As an intervention strategy they received fencing to prevent their livestock from roaming on and destroying areas where crops are being cultivated. Because men could use their lands more productively, they demonstrated a clear value of group memberships to other men, their families and the wider community. Their gained sense of usefulness meant that they no longer needed to compensate their perceived ‘unmanliness’ through violent and dominant behaviour.

#### Affective level: opening up safe space for reflection

Emotions play a key role in transforming masculinities [26]. Men who are emotionally engaged in the intervention content are more likely to interrogate their own complicit role of oppression and recognise their responsibility to change [4, 55].

Learning to show more emotions can significantly improve relationships as this 41-year-old OMC participant explained:

*I believed in the old way of doing things that a man should not show his emotions or love openly but OMC changed me because now I can show my girlfriend how much I love her.* [45], p. 1030)

On a practical level, studies argue for the importance of safe spaces that allow emotional expression and vulnerability amongst participants and are an important means to challenge and reflect upon destructive masculine behaviours [52]. The different interventions have varying understandings of what a safe space might look like in practice. Whereas Jobson [52] and Keddie [55] for instance regard safe spaces as a place to discuss sensitive and taboo issues without judgment, Gibbs and colleagues [38, 39] were more critical of safe spaces and argue that competition between different forms of masculinity was the norm in the interventions and that safety was only really established in those sessions that were particularly emotionally charged. Similarly, Keddie [55] highlights the unavoidable occurrence of discomfort when unsettling taken-for-granted views and assumptions, both on the facilitator and the participant side and advocates for critically reflecting on the various emotional intensities at play. This is especially true when facilitators ‘call out’ unacceptable behaviour around topics of consent and ownership of one’s own body. Facilitators then need to find a balance between confrontation and participatory conscious building. Those interventions vary in approaches ranging from a corrective confrontation [76] to more nuanced, context-dependent challenges and emphatic and respectful question-raising [55].

#### Cognitive-behavioural level: knowledge as power

A main component of most interventions was educational content. This includes factual information about male and female biology, sexuality and sexual practices, explanations of how gender is socially constructed, power dynamics and privileges, identifying different types of violence, bystander intervention, contraception options or specific information about HIV (see for example: [4, 32, 52, 53, 85]. The extent and depth of this education varied widely between different programmes and spanned a three-year duration [79] and 100 h of course content in the form of a diploma course [4] to a delivery of four one-hour sessions [8]. Content can be delivered in the form of a curriculum and delivered by professionals or peers but it can also be self-directed and online [86].

A change in knowledge may also entail a change in power relations, as decisions relating to consent and sexual intercourse, for instance, can be made from an informed standpoint about reproductive risks and rights. Having learned about appropriate condom use, one participant’s comments in an India-based adaptation of Programme H (in [85], p. 139) shows how better knowledge about sexuality and bodies can also improve relationships and make them possibly more equitable: “It’s not only penetrative sex but every part of our body that can give us sexual pleasure. Understanding between partners is the main thing’’.

Interventions typically sought to create a social learning environment in the form of facilitator-led dialogues that helps participants reflect on and apply the learned content. It is in these environments, where participants may translate their learnings into concrete actions, e.g. stopping to smoke, reducing violence [45], p. 1031) or reducing alcohol consumption which is also linked to reduction of violent behaviour, improved condom use, and thus HIV prevention [39, 45, 81]. However, other studies indicate that a change in behaviour is not always congruent with cognitive shifts. Sometimes participants might act less violently, but struggle to absorb and accept lessons concerning gender equity [12, 76].

#### Community-structural level: beyond individual level transformation

This level is concerned with the question *who* the interventions actually target and view ‘in need’ of transformative intervention. To some degree this depends on the health focus of the intervention and the breadth and reach of the target population. In interventions that work directly with adult male perpetrators of violence against women (see for instance [12, 76], men may be stigmatised as generally dangerous, without self-constrain and criminal which might lead to further stigmatisation. If transformation occurs it is often just within the behaviours of specific individuals, while gender relations on a wider level remain unchanged. As Kato-Wallace and colleagues [53] note when working in neighbourhoods struggling with systemic racism, poverty, inequitable distribution of resources, and high levels of violence, it is unclear if individual-level changes in knowledges, attitudes and behaviours can have substantial enough impact on structural inequalities.

Violence prevention programmes in schools aimed at young men and boys [8, 64] already take a wider scope in that they critically engage with relations amongst boys, and between boys and girls; thereby widening the focus onto small groups, including sport clubs and classrooms [31, 32].

Namy and colleagues [66] emphasise that gender-transformative approaches ought to be gender synchronised, that is, addressed at and engaging both girls and boys equally. The authors are critical of approaches that engage with only boys and men (S219).

Interventions aimed at violence prevention that engaged with both men and women, like OMC and the Man’s Story Project, took an even broader scope in that they considered fathering roles and put into focus experiences of gay, disabled, and marginalised men [33, 45, 68].

Yet other programmes, like those based on Program H, regularly undertake a community asset mapping at the beginning of the intervention and are thus able to involve multiple and diverse community stakeholders, such as churches and sports clubs (see for instance [17, 85]. Livelihood interventions like SFHC and an unnamed one run by the Valley Trust also actively grapple with local stakeholders from the economy while seeking to transform household roles [52].

One unusual intervention in Colombia [4] intervened directly on a community and structural level and targeted their intervention at civil servants, both men and women, who ought to reflect on how they perpetuate and challenge gender stereotypes in their personal relationships and at work. We regard these as more structural as they concern people in positions of power, whose decisions and behaviour affect a broad societal spectrum.

## Discussion

We first make some general observations about the ways that masculinities are conceptualised and operationalised. We then move on from treating theory and practice as though they were two separate domains, and, through the lens of feminist philosophy of science, focus on ways to translate theory into practice. Third, we consider the performative function that the choice of theory may have and highlight ethical implications. Fourth, we shift the focus to learnings for GTIs in general. Finally, we discuss strengths and limitations of this review.

### General observations about the ways masculinities are conceptualised and operationalised

We examined how masculinities were conceptualised and operationalised in theory and practice of GTIs. Overall, masculinities tended to be conceptualised through the lens of hegemonic masculinities. Modes of operationalisation varied between being very narrow and specific, to being quite wide and ecological in focus. The mechanisms through which masculinities were operationalised in theory and practice were heterogenous and heavily depended on the specific aims, context, and design of the intervention itself. We can also assume that the ways masculinities were conceptualised and operationalised also depended on the institutional backgrounds and training of the authors and intervention practitioners. Here specific conceptualisations of masculinity might depend on what degree they are affiliated with a specific academic community, expectations and conventions of that academic community, and expectations of funding bodies and other intervention stakeholders.

Longer-term interventions with the aim to learn more informally and reflect in flat hierarchies tended towards critical pedagogic frameworks (such as OMC, Promundo, BTM, or MSP), while interventions that were more short-term and about delivering a structured curriculum, such as those taking place in schools and prisons (such as WiseGuyz, YBMen, or CMIB), were interested in theories of social influence. However, at this point, we cannot make a generalisable observation or identify a pattern as to what kinds of interventions lean towards what kinds of conceptualisation or operationalisations of masculinity. The lack of pattern speaks to the multiplicity of approaches, versatility of application, and adaptability to context of ‘masculinities’ in GTIs [50, 51].

### Translating between theory and practice

We consider conceptualisation and operationalisation, theory and practice, as parts of a whole process. Hence this review aims to identify how theory and practice link together and can be translated from one to another. Some of these translations between theory and practice map out quite clearly, for instance, how affect theory also describes the role of emotions in transformative processes within the intervention. Other intervention studies seem to struggle with making the transition between theory and practice. For instance, in our review the material level of interventions has no direct translation within theoretical frameworks, even though it seems to play an important role in changing the local gendered economy, which in turn has concrete outcomes in terms of household roles and violence reduction. Within more feminist and relational approaches to health there is a growing body of theory—new materialism— which posits, in very simple term, that matter has an agentive force on social processes of change [3]. Such approaches bear great potential for theoretical advancements within gender-transformative research and practice.

We suggest here that gender-transformative scholars and intervention practitioners ought to continue to ground their practices within diverse, novel and emergent frameworks that push gender and masculinities theories into new applications and change-making mechanisms. At the same time, we also need to acknowledge that theory, especially that developed in a feminist vein, can be “messy” and “weak”- these terms are not meant derogatory in the literature but to the contrary highlight the importance of acknowledging the fluid, relational co-dependencies of social worlds; in other words, no *one* theory can neatly describe a reality [2, 42]. Theory should therefore avoid building ‘grand’ or ‘essentialising’ narratives, or ‘one size fits all’-approaches that aim to explain the world in total. The ‘job’ of theory, understood in a “messy” and “weak” feminist vein is thus to acknowledge how we are mutually and plurally entangled in our environments in a specific context, and only ever situated and limited in our understanding of it.

In the context of GTIs, we see great potential for a grounded theory approach, an approach which builds theory bottom-up from the narratives that emerge out of research data, rather than trying to prove or disprove already formulated hypothesis. This could be coupled to the initial research phase, such as in the Promundo interventions, that informs intervention design but could perhaps also be analysed so to inform theory design.

Thus, when translating theory to practice we advocate for theoretical pluralism and theoretical innovation with the aim to continue heterogenous and non-essentialising discourses which are grounded in practice. One way to achieve this aim might be through interdisciplinary collaborations between the humanities, social science, and public health research, as well as cross-sectoral collaborations between relevant community and voluntary sectors, health organisations, individual intervention practitioners and academics.

### Theory, performativity and ethics

Simply using more or other theories to describe various social realities, however, is not enough when it comes down to leveraging social change through research and practice. In this third discussion point, we highlight the limits of masculinities as a concept and the ethical impetus behind choosing theoretical frameworks. In the context of gender-transformative HIV care, [19], p. e192) cautions that although, as a theory, masculinity and hegemonic gender norms are a useful and powerful tool for analysis, overuse of this concept should be avoided when trying to identify ways to move to equity. HM as a concept is thus rather descriptive of *what is*, but stalls at pointing to *what could/should be* [50, 51]. In our review, we identified six wider theoretical approaches within the nexus of masculinities and gender-transformation. These various approaches helped operationalise and transform masculinities analytically by up-scaling and projecting individual level behaviour and attitudes onto larger frames of analysis which can speak to structural shifts in gender norms and relations.

Each of these theories is grounded in its own socio-historical scientific practices that each bring their own geographies, nuances, and semantics to describing processes of change. As an example, critical pedagogy emerged from post-Marxist, post-colonial educational praxis in Brazil. It builds on educational principles that transform masculinities not by simply instilling knowledge on how to be a ‘good man’, but by questioning one’s own positionality and power, dispelling cultural myths, and calling out structures of oppressions by ways of feeling, thinking, and acting in power-conscious manners. Similar to theories of social influence, critical pedagogy tries to explain *how exactly* thinking differently leads to acting differently, how changed attitudes change behaviour, changed behaviours change actions, and real-life actions change socio-political systems [79].

Feminist philosophers of science have long argued that theory is not something merely descriptive or abstract but that is has a performative function, that means *it enacts the worlds it describes*, gradually and over time [42, 88]. This review thus made clear that the choice of theoretical frameworks is not a random act of academic fancy with random consequences. Choosing theory is something researchers can do consciously depending on the discourses they speak to and the changes they hope to achieve with their intervention. Barad [9], another feminist philosopher of sciences, therefore argues that ontology (the assumptions that research makes about how the world *is*) and epistemology (how we come to *know* about the world) is inextricably connected to ethics, and considerations on what a kind of world we would like to promote.

We wonder to what extent researchers might broaden their understanding of what an ‘ethical research field’ is and include the possibly undervalued ethical and political impact that the choice of theory can have on participants, practitioners and policy makers. Thus, understanding processes of gender-transformation and transformation more widely also requires an ethical and political engagement with theories of change and transformation, some of which we have identified in this review.

### What we can learn about GTIs in general: implications for future developments

The socio-demographic, geographical and epidemiological contexts of the studied interventions in this review are heterogenous in nature. Moreover, the intervention studies addressed multiple health foci and often highlighted interdependent, syndemic and relationally situated clusters of health and wellbeing of communities. One key learning is that one size does *not* fit all as each intervention generates different challenges, questions, and pathways, each offering different options to theoretically and practically engage with.

What almost all our interventions had in common though is that they tended to take a preventative approach to health, rather than focussing on a single disease or a single group of individuals. They did so, for instance, by focussing on capacity-building and building healthy masculinities [8, 86]. We also note an absence of formal healthcare settings within the interventions, and notice the prevalence of informal, community-based settings of health care. One exception are interventions focussing on HIV/AIDS care where testing and knowing one’s status were an important part of the intervention [33]. Yet, it could be argued that GTIs differ from traditional health frameworks that focus on diagnosis and disease treatment of the individual within traditional biomedical and more formal care settings. In a review singling out factors for successful gender-transformative programmes, Heymann and colleagues [47] identified multisectoral action, multilevel and multistakeholder involvement, and diversified programming as three of the four main factors underpinning sustainable change towards gender equity. This has also become clear in interventions of this review that highlight the role that local contexts and community networks play in carrying out GTIs [36]. GTIs thus play a key role in a wider trend in health policy and practice that advocates for a more preventive, ecological and community-centred model for health, which next to being effective in improving health outcomes also tends to be much more cost-effective in comparison to individual and acute care within formal health delivery settings.

This begs the question if more formal care settings could also be enhanced through community-driven gender-transformative programmes that engage with masculinities. It also raises the question in what other areas of health GTIs with a focus on masculinities might be conducive to positive outcomes. So far, the majority of GTIs in this review focus on gender-based violence or sexual health; other promising but to date under-researched applications of a GTI/masculinity lens could include peri-natal health care (athough see [18] or seemingly more far-fetched applications like lifejacket use and injury and drowning prevention [74].

### Strengths and limitations

The review is the first scoping review to map how masculinities are conceptualised and operationalised across a wide spectrum of heterogenous GTIs. As such we offer broad but transferable and actionable insights relevant for the future of the field of both, masculinities and GTIs. Our search strategy helped identify two lesser-known interventions not published in English, which provided relevant and diverse learnings from the Global South, although we were dependent on an AI-based translation system which might not have caught linguistic nuances. There are other limitations to our review. First, we only searched for studies that describe themselves as gender-transformative in at least one component of the intervention. It falls outside the remit to assess whether these studies were really transformative as definitions remain fuzzy in this still emergent field of research and practice. Equally, we might have missed studies that were in their nature gender-transformative but whose authors did not describe them as such or were not familiar with this particular framework. Our review is somewhat restrained by academic conventions and language emerging out of the field of public health literatures and thus might leave out relevant observations from other academic disciplines. This relates to a second limitation, namely that interventions strictly focused on, for instance, criminal justice or education were not included despite their possible but not reported impact on people’s health. Thirdly, we also acknowledge that although two researchers reviewed and analysed the literature, our judgment, especially in relation to what ‘theory’ needs to ‘look like’, remains somewhat subjective and influenced by our institutional and cultural backgrounds (JZ and SBZ are both trained as sociologists at universities in the Global North). Finally, because we only included peer-reviewed literature, a large body of possibly relevant theoretically-informed grey literature remains unaddressed in this review.

## Conclusion

Engaging men in GTIs for health equity is clearly important, yet there was previously no synthesis of the ways masculinities were conceptualised and operationalised in GTIs. Our review fills this gap. It highlights the heterogeneity of approaches within theory and practice and shows how masculinities, as a concept, are highly adaptable to various settings and frameworks. In this scoping review we have: i) identified how different version of hegemonic masculinities are evident within specific intervention settings, ii) delineated five different theoretical approaches that embed masculinities within wider approaches towards social change and transformation, and iii) mapped out six different levels on which transformations may be observed within the interventions, as reported in the outcomes of the included studies. We identified possible applications and theoretical frameworks within the nexus of GTIs/masculinities; they, however, also highlight that one size does *not* fit all. Thus, the discussion makes a point for theoretical pluralism and innovation and highlights the ethical implications of theory choice. Because of the high heterogeneity and adaptability of operationalisation mechanisms between GTIs and masculinities, we see great potential in applying and developing this lens in a plurality of health care settings, towards the improvement of a broad range of health outcomes.

## Data Availability

Not applicable/Data sharing is not applicable to this article as no datasets were generated or analysed during the current study.

## Appendix 1

### Search terms

("gender sensitive"[Title/Abstract] OR "gender-sensitive"[Title/Abstract] OR "gendersensitive"[Title/Abstract] OR "gender transformative"[Title/Abstract] OR "gender-transformative"[Title/Abstract] OR "gendertransformative"[Title/Abstract] OR “gender transformation”[Title/Abstract] OR "gender-responsive"[Title/Abstract] OR "gender responsive”[Title/Abstract] OR "genderresponsive"[Title/Abstract]OR “gender specific”[Title/Abstract] OR “gender-specific”[Title/Abstract] OR “genderspecific”[Title/Abstract] OR “gender-equality”[Title/Abstract] OR “ gender equality”[Title/Abstract] OR “gender-equal”[Title/Abstract] OR “gender equal”[Title/Abstract] OR “gender equity”[Title/Abstract] OR “gender-equity”[Title/Abstract] OR “gender- equitable”[Title/Abstract] OR "challenging gender norms” [Title/Abstract] OR “challenging gender attitudes” [Title/Abstract] OR “challenging gender roles”[Title/Abstract] OR "changing gender norms”[Title/Abstract] OR “changing gender attitudes”[Title/Abstract]OR “changing gender roles” [Title/Abstract] OR "disrupting gender norms”[Title/Abstract] OR “disrupting gender attitudes”[Title/Abstract] OR “disrupting gender roles”[Title/Abstract]) AND ("masculinity"[Title/Abstract] OR "masculinities"[Title/Abstract] OR "machismo"[Title/Abstract] OR "masculine gender norms"[Title/Abstract] OR "male gender norms"[Title/Abstract] OR "male identity"[Title/Abstract] OR "manhood"[Title/Abstract] OR "boyhood"[Title/Abstract] OR "manliness"[Title/Abstract] OR "male-sensitive"[Title/Abstract] OR "male-friendly" [Title/Abstract]).
